# Speed Measurement of the Moving Targets Using the Stepping Equivalent Range-Gate Method

**DOI:** 10.3390/s24051437

**Published:** 2024-02-23

**Authors:** Gang Yang, Zhaoshuo Tian, Zongjie Bi, Zihao Cui

**Affiliations:** Institute of Marine Optoelectronic Equipment, Harbin Institute of Technology at Weihai, Weihai 264209, China; 16b921025@stu.hit.edu.cn (G.Y.); bizongjie@hit.edu.cn (Z.B.); cui_zh@hit.edu.cn (Z.C.)

**Keywords:** range-gated, imaging lidar, moving target, 3D imaging

## Abstract

In this paper, we proposed a stepping equivalent range-gate method (S-ERG method) to measure the speed and the distance of the moving target for range-gated imaging lidar. In this method, the speed is obtained by recording the time at which the moving target passes the front and back edges of the range gate, the distance information can also be obtained by the front and back edges of the range gate at the same time. To verify the feasibility of this method, a stationary target and a moving target with different speeds were measured by the S-ERG method. By using the S-ERG method, we not only obtained the distance information of the stationary target and the moving target at the front and back edges of the range gate, respectively, but also obtained the speed of the moving target. Compared to speeds measured by rotational displacement sensors, the speed measurement error of the S-ERG method is less than 5%, whether the target is far away or close to the range-gated lidar system, and this method is almost independent of the delay step time. The theoretical analysis and experimental results indicate range-gated imaging lidar using the S-ERG method has high practicality and wide applications.

## 1. Introduction

According to the different ways of acquiring three-dimensional (3D) information about the target, the imaging lidar can be divided into point imaging scanning lidar, line scanning imaging lidar, and surface array imaging lidar [1]. The point-scanning lidar and line-scanning lidar are collectively referred to as scanning lidars, and both of them require the laser transmitter system to be mounted on a more complex scanning mechanism. Among them, point-scanning lidar generally uses a single-point detector, which can obtain the distance information of one pixel point per measurement, and achieve the target distance information within a certain range through the scanning system; line scanning lidar, on the other hand, uses galvanometer, push sweep and other methods to cover the laser light, which is rectified into a line shape, to the target, and complete the acquisition of the distance information of the target. Therefore, although the scanning lidar has the advantages of high imaging pixels and high distance resolution, the complex mechanical scanning structure seriously limits the imaging frame rate, imaging quality and imaging speed of the scanning lidar [2,3,4], and at the same time, the scanning system causes serious aberrations in the distance information of the target acquired by the lidar [5], and more complex algorithms are required to eliminate the aberrations, which greatly restricts the application of the scanning lidar. In contrast, the array imaging lidar uses a two-dimensional detector array as a receiver to simultaneously acquire a large amount of 3D information on the target surface without waiting for an acquisition time proportional to the number of pixels to be measured, which enables fast 3D detection with large resolution. Therefore, compared with the scanning lidar system, the advantages of surface array imaging lidar, such as small size, fast imaging frame rate, high resolution, etc., make it become a research hot spot at home and abroad [6,7,8,9].

Range-gated technology is widely used in array imaging lidar systems, which uses a controllable pulse laser and a selective intensified-charge-coupled device (ICCD) that can be opened and closed at high speed to separate the reflected and backscattering light from targets at different distances [10,11] so that the laser pulse signals reflected from the target to be measured will reach the camera and be imaged in exactly the selective time of the ICCD. During lidar imaging, poor imaging quality is due to backward-scattering by atmospheric, water and other media in the optical path. The range-gated technique can be used to obtain target intensity images at different distances by varying the delay time, thus eliminating most of the backscattering light and background noise effects. In this way, only the light signal reflected by the target can enter the receiving system of the lidar; thus, the signal-to-noise ratio (SNR) of the 3D image will be improved [12].

Although frequency-modulated-continuous-wave (FMCW) lidar can be useful for enabling the measurement of the velocity of moving targets [13,14,15,16], compared to range-gated lidar, it has a smaller measuring range. When a range-gated imaging lidar system with a traditional 3D reconstruction method (such as time-slicing method [17], the trapezoid-range-intensity profile method [18], the triangular-range-intensity profile method [19] and the centroid method [20]) is used to measure a moving target, it is not easy to realize the measurement of the moving target because the target is in a moving state, which seriously limits the application of imaging lidar. In this paper, based on the feature that the adjacent frame method (AFD method) can obtain the target distance information at the front and back edges of the range gate, respectively, the equivalent range-gate method (ERG) is proposed, with which not only the distance information of the moving target and the stationary target can be obtained, but also the speed and direction of the moving target can be measured.

## 2. Methods

The AFD method is a new 3D reconstruction algorithm based on the range-gated method [21]. In this method, the distance information of the target at the front and back edges of the range gate is obtained by thresholding the intensity difference between two adjacent frames achieved by the ICCD.

### 2.1. Statics ERG Method to Measure the Speed of the Moving Target

**Figure 1:**
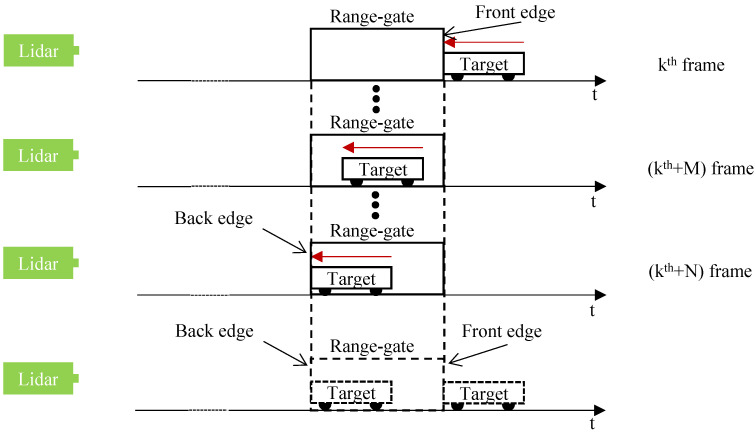
When the target moves towards the lidar system, a schematic diagram of the speed measurement of a moving target using the static ERG method.

The ERG method is based on the AFD method. When the target moves towards the lidar system, a schematic diagram of the speed measurement of a moving target using the static ERG method is shown in Figure 1.

In Figure 1, the red arrow indicates the direction of movement of the target. As range-gated imaging lidar works, the pulse laser system is triggered by the signal generator. The laser emits a pulse with a pulse width of $\tau_{p}$ to the target and triggers the time delay device simultaneously. For each trigger, the delay time is increased by one step as $\Delta \tau_{step}$. Assuming the $k^{\mathrm{th}}$ laser pulse just reaches the target, the image acquired by the ICCD is recorded as the $k^{\mathrm{th}}$ frame with the corresponded delay time of the range gate as $k\Delta \tau_{step}$. The intensity of the pixels in this $k^{\mathrm{th}}$ frame corresponding to the target is expected to be high, defined as $I_{\left(m,n\right)}^{k}$, (m,n) is the coordinates of each pixel. Considering one frame before the $k^{\mathrm{th}}$ frame as the ${k-1}^{\mathrm{th}}$ frame, since the pulse laser corresponding to this frame does not reach the target, the intensity of the pixels in this frame corresponding to the target should be low and similar. In the AFD method, a new image is first generated based on two adjacent frames. In this new image, the intensity value of each pixel is the difference between the corresponding pixels in the two adjacent frames. Then, the values of these pixel intensity differences, defined as ΔI, would be compared with the intensity threshold δ. Normally, we set this threshold to one-fifth of the max pixel intensity value. At the front edge, the δ is a positive value and marked as $\delta^{+}$, which is used to verify the validity of pixels at the front edge. At the back edge, the δ is a negative value and marked as $\delta^{-}$, which is used to verify the validity of pixels at the back edge. Based on the $k^{\mathrm{th}}$ and ${k-1}^{\mathrm{th}}$ frames, the intensity of each pixel corresponding to the target on the newly generated image should be larger than the threshold, as $\Delta I_{\left(m,n\right)}^{k}>\delta^{+}$. Thus, the pixels corresponding to the target in this newly generated image are valid. Meanwhile, since these pixels in this image correspond to the front edge of the range gate, the moving target appears at the front edge of the range gate.

With the moving of the target, assuming that in the ${k+N}^{\mathrm{th}}$ frame, the moving target appears at the back edge of the range target. This means that the moving target moved exactly the distance corresponding to one range gate during the time corresponding to the ICCD acquisition of N frames. Supposing the target is moving at a constant speed during this time, the speed of this target can be expressed as:

(1) $$v=\frac{(\Delta \tau_{\mathrm{gate}}\times c)\times u}{2N},$$

where *v* is the speed of the moving target, *c* is the light speed, *u* is the imaging frame rate of the lidar system and *N* is the difference in the number of frames corresponding to when the target is observed at the front and back edges of the range gate, respectively.

**Figure 2:**
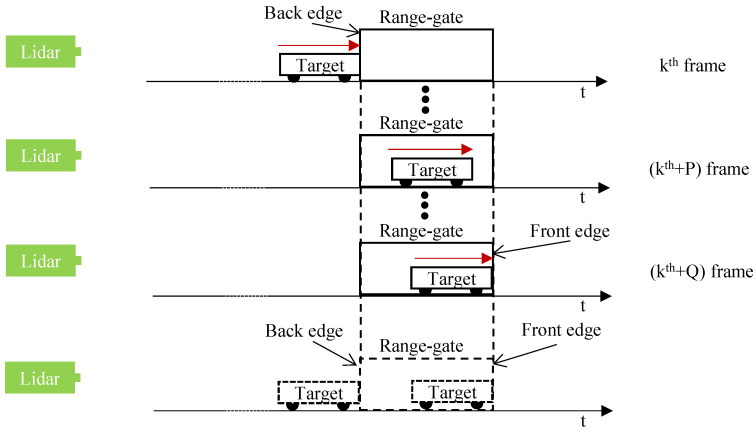
When the target moves away to the lidar system, a schematic diagram of the speed measurement of a moving target using the static ERG method.

As the target moves away from the lidar system, a schematic diagram of the speed measurement of a moving target using the static ERG method is shown in Figure 2.

In Figure 2, the red arrow indicates the direction of movement of the target. Assuming that in the $k^{\mathrm{th}}$ frame of the intensity image, the moving target just appears at the back edge of the range gate; in the $k\;+\;Q^{\mathrm{th}}$ frame of the intensity image, the moving target just appears at the front edge of the range gate. Then, it is stated that the target has moved by exactly one distance corresponding to a range gate during the time experienced in the acquisition of the intensity image of frame $k^{\mathrm{th}}$ and the intensity image of frame $k\;+\;Q^{\mathrm{th}}$. Suppose the target is moving at a constant speed during this time, the speed of this target can be expressed as:

(2) $$v=\frac{(\Delta \tau_{\mathrm{gate}}\times c)\times u}{2Q},$$

From Figure 1 and Figure 2 and Equations (1) and (2), when measuring a moving object, the width of the range gate should be larger than the length of the object to be measured; otherwise, the target object will appear at both the front and back edges of the range gate.

### 2.2. Stepping ERG Method to Measure the Speed of the Moving Target

Although the speed of a moving target can be measured using the static ERG method, but it is difficult to obtain the distance information of the moving target. Therefore, three-dimensional information of the target in different distance ranges and the speed of the moving target can be obtained by stepping the equivalent range-gate method (S-ERG method).

**Figure 3:**
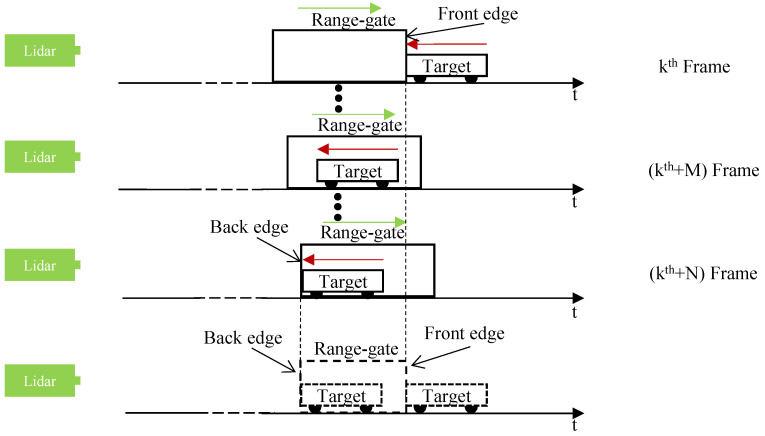
When the target moves towards the lidar system, a schematic diagram of the speed measurement of a moving target using the static S-ERG method.

When the target moves towards the lidar system, a schematic diagram of the speed measurement of a moving target using the static ERG method is shown in Figure 3.

In Figure 3, the red arrow indicates the direction of movement of the target, the speed is *v*; the green arrow indicates the direction of the stepping range gate, the step size is $\Delta \tau_{step}$. Assuming that in the $k^{\mathrm{th}}$ frame of the intensity image, the moving target just appears at the front edge of the range gate; in the ${k+N}^{\mathrm{th}}$ frame of the intensity image, the moving target just appears at the back edge of the range gate. Due to the relative motion of the target and the stepping range gate, the equivalent range gate is compressed in time, and assuming that the size of the compression is $\Delta \tau_{\mathrm{gate}}^{\mathrm{comp}}$, so:

(3) $$\Delta \tau_{\mathrm{gate}}^{\mathrm{comp}}=\Delta \tau_{\mathrm{step}}\times N,$$

the speed of the moving target is:

(4) $$v=\frac{c(\Delta \tau_{\mathrm{gate}}-\Delta \tau_{\mathrm{gate}}^{\mathrm{comp}})u}{2N},$$

Combining Equations (3) and (4), the speed of the moving target measured by S-ERG method is:

(5) $$v=\frac{c(\Delta \tau_{\mathrm{gate}}-N\Delta \tau_{\mathrm{step}})u}{2N},$$

At the same time, the distances corresponding to the moving target at the front and back edges of range gate are $d_{towards}^{front}$ and $d_{towards}^{back}$, respectively. They can be expressed as:

(6) $$d_{towards}^{front}=\frac{ck\Delta \tau_{\mathrm{step}}}{2},d_{towards}^{back}=\frac{c\left(k+N\right)\Delta \tau_{\mathrm{step}}}{2},$$

**Figure 4:**
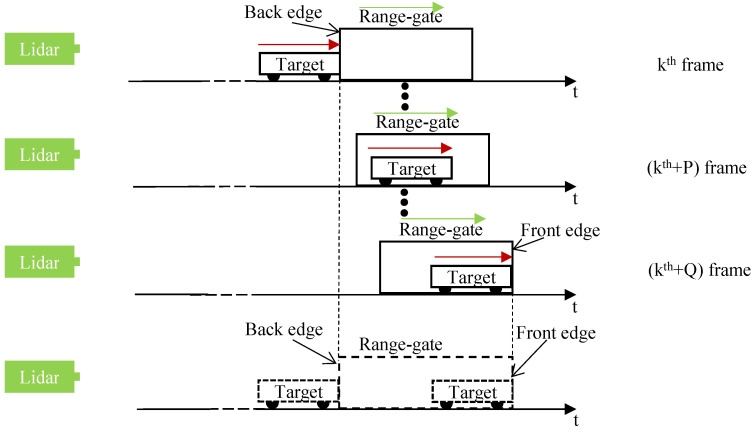
When the target moves away to the lidar system, a schematic diagram of the speed measurement of a moving target using the static S-ERG method.

When the target moves away to the lidar system, a schematic diagram of the speed measurement of a moving target using the static ERG method is shown in Figure 4.

In Figure 4, the red arrow indicates the direction of movement of the target, the speed is *v*; the green arrow indicates the direction of the stepping range gate, and the step size is $\Delta \tau_{step}$. Assuming that in the $k^{\mathrm{th}}$ frame of the intensity image, the moving target just appears at the back edge of the range gate; in the $k\;+\;Q^{\mathrm{th}}$ frame of the intensity image, the moving target just appears at the front edge of the range gate. Due to the relative motion of the target and the stepping range gate, the equivalent range gate is widened in time, and assuming that the size of the widen is $\Delta \tau_{\mathrm{gate}}^{\mathrm{widen}}$,

(7) $$\Delta \tau_{\mathrm{gate}}^{\mathrm{widen}}=\Delta \tau_{\mathrm{step}}\times Q,$$

the speed of the moving target is:

(8) $$v=\frac{c(\Delta \tau_{\mathrm{gate}}+\Delta \tau_{\mathrm{gate}}^{\mathrm{widen}})u}{2Q},$$

Combining Equations (7) and (8), the speed of the moving target measured by S-ERG method is:

(9) $$v=\frac{c(\Delta \tau_{\mathrm{gate}}+Q\Delta \tau_{\mathrm{step}})u}{2Q},$$

At the same time, the distances corresponding to the moving target at the front and back edges of range gate are $d_{away}^{front}$ and $d_{away}^{back}$, respectively. They can be expressed as:

(10) $$d_{away}^{back}=\frac{ck\Delta \tau_{\mathrm{step}}}{2},d_{away}^{front}=\frac{c\left(k\;+\;Q\right)\Delta \tau_{\mathrm{step}}}{2},$$

So, based on Equations (5), (6), (9) and (10), the speed information and the distance information of the moving target can be measured by the S-ERG method.

The only difference between the ERG method and the S-ERG method is that the delay time of the range-gate signal in the ERG method is fixed, while the delay time of the range-gate signal in the S-ERG method is increased in steps. Therefore, the ERG method can only measure the speed of the moving target, while the S-ERG method can not only measure the speed of the moving target, but also obtain the distance information of the moving target.

## 3. Experiment

### 3.1. Speed Measurement by Static ERG Method

**Figure 5:**
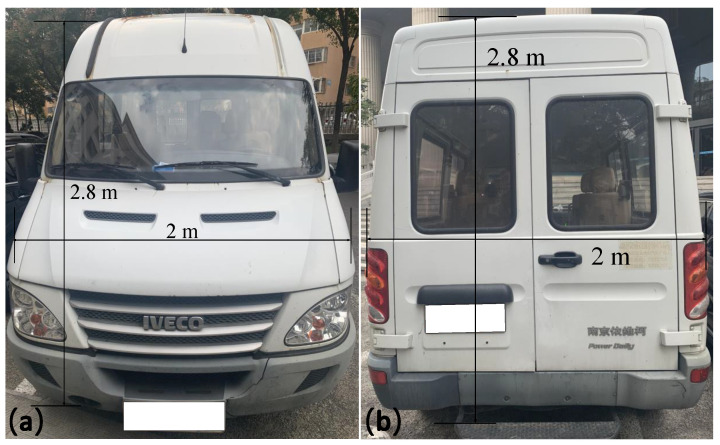
(**a**) Front view of the moving target. (**b**) Back view of the moving target.

The moving target is an IVECO car with a length of 5.5 m, 2 m wide and 2.8 m high, as shown in Figure 5. In order to accurately measure the speed of a moving target, the width corresponding to the range gate should be larger than the length of the target, therefore, the width of the range gate was set to 100 ns in this experiment.

The experiment was carried out using the self-developed underwater imaging flash lidar system. A Pilot-3 air-cooled pulsed laser with a center wavelength at 532 nm is used as the irradiation source with the 5 ns pulse width and the 1 kHz pulse repeat frequency, respectively. The averaged energy per pulse is 2 mJ. The laser divergence angle is adjusted by a collimation system. The size of the ICCD is 1292×964, the bit-depth of the ICCD is 8. When the laser emits a pulse laser, the trigger delay system starts timing, and the delay time and range-gate width are set by the time delay controller to generate a precisely delayed trigger signal. This range-gate signal is further used for triggering the ICCD to realize the selection of the imaging area, and to acquire the laser signal reflected by the target.

Speed measurements were taken for the case of the moving target approaching and moving away from the imaging lidar system, respectively. Six different sets of speeds were randomly collected for each case, and the measurements are shown in Table 1 and Table 2, the actual speeds were measured by rotational displacement sensors.

Whether the moving target is close to the lidar system or the is far away from the lidar system, compared to speeds measured by rotational displacement sensors, the static ERG method is a highly accurate way to measure the speed of the moving target with max error of 5.39%.

### 3.2. Speed Measurement by S-ERG Method

**Figure 6:**
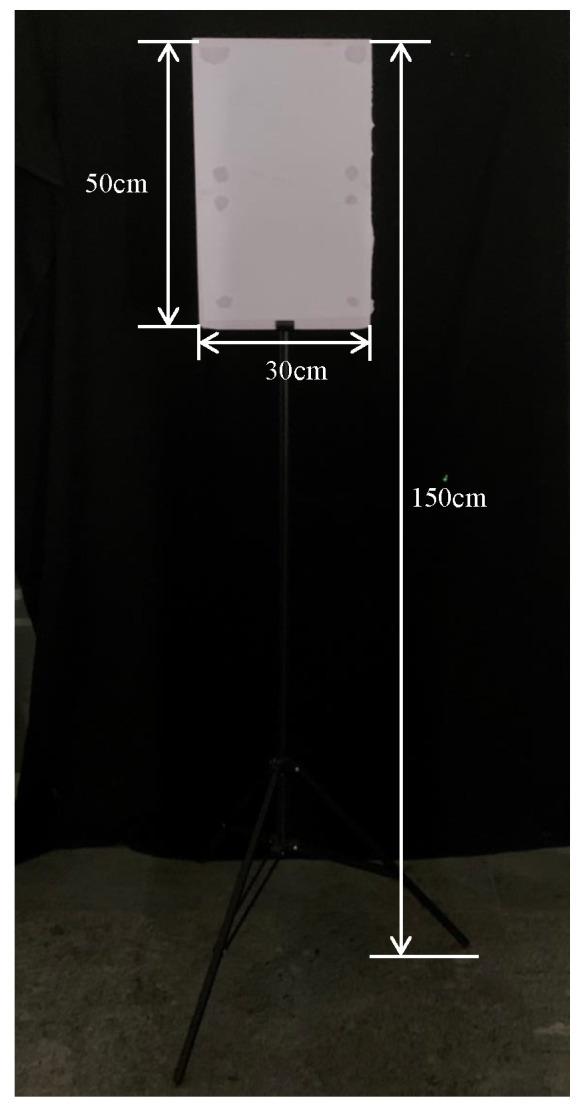
Stationary target.

In order to validate the feasibility of the S-ERG method, we have measured both moving and stationary targets. The moving target is shown in Figure 5. The stationary target is a rectangular whiteboard with a length of 50 cm, a width of 30 cm, and is placed on a stand about 1.5 m high. As shown in Figure 6.

Velocimetry experimental studies were first carried out on targets moving towards the imaging lidar system by setting the delay step size of 1 ns and 5 ns, respectively. The measurement results are shown in Table 3, and the actual speeds were measured by the rotational displacement sensors.

From Table 3, it can be seen that the accuracy of the results is high when the delay step is 1 ns or 5 ns, which fully demonstrates that the S-ERG method can accurately obtain the speed of the moving target, and the selection of delay step size has little influence on the S-ERG method. However, according to the AFD method, the shorter the delay step size is set, the more accuracy of the target distance information is obtained. Therefore, in the subsequent experiments, the delay step size was set to 1 ns.

Comparing Table 1 with Table 3, the accuracy of the ERG method to obtain the target moving speed is a little bit higher. This is due to the fact that in the ERG method, the delay time of the range gate signal is constant, whereas in the S-ERG method, the delay time of the range-gate signal is increased in steps, which increases the jitter of the delay signal and reduces the accuracy of the measured velocity. However, the S-ERG method can not only obtain the moving speed of the moving target, but also the distance information of the target.

**Figure 7:**
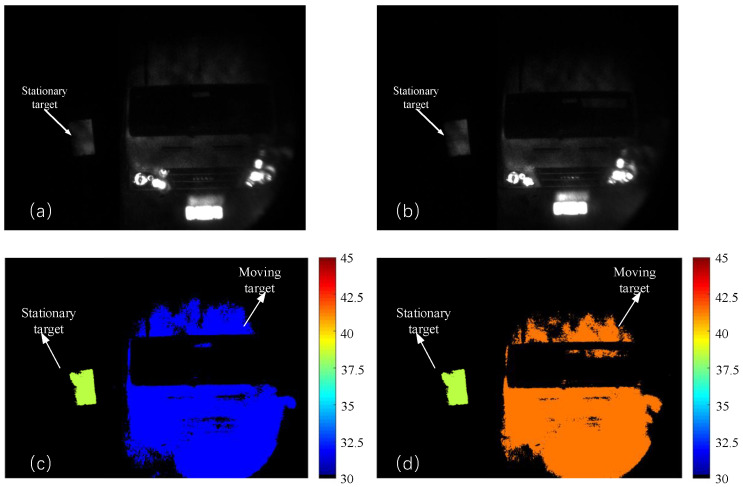
The step size is 1 ns. (**a**) Intensity image at front edge of the range gate. (**b**) Intensity image at back edge of the range gate. (**c**) Distance image at front edge of the range gate. (**d**) Distance image at back edge of the range gate. The units of the colored figures are ’meters’. The speed of the moving target is 18.44 km/h measured by the S-ERG method.

When the moving target is moving towards the LiDAR system and the speed is 17–18 km/h, the distance images corresponding to the front and back edges of the selector gate obtained by this method are shown in Figure 7.

From Figure 7c,d, as the moving target approached, its color gradually changes from red, representing distance, to blue, representing proximity, and there is no change in the color of the stationary target.

When the target was moving away from the imaging lidar system, six sets of random speed measurements were taken on the moving target with a delay step size of 1 ns, and the obtained target speeds are shown in Table 4; the actual speeds were measured by the rotational displacement sensors.

**Figure 8:**
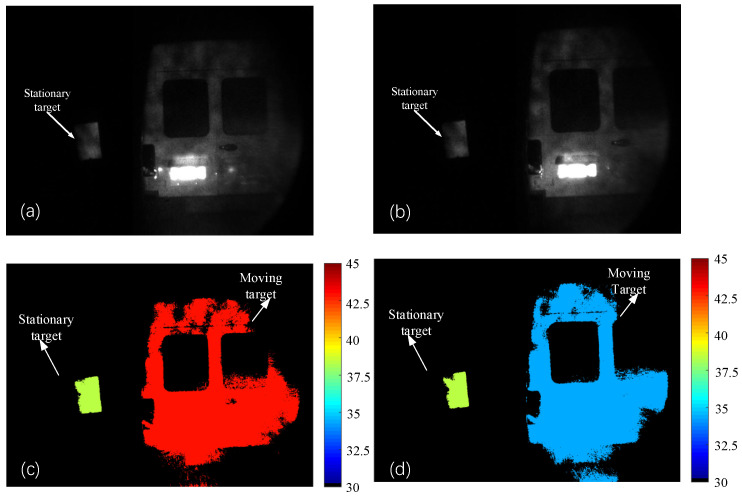
The step size is 1 ns. (**a**) Intensity image at the front edge of the range gate (**b**) Intensity image at the back edge of the range gate. (**c**) Distance image at the front edge of the range gate. (**d**) Distance image at the back edge of the range gate. The units of the colored figures are ’meters’. The speed of the moving target is 18.50 km/h measured by the S-ERG method.

When the moving target is moving away from the lidar system and the speed is 18–19 km/h, the distance images corresponding to the front and back edges of the range gate obtained using the S-ERG method are shown in Figure 8. As can be seen in Figure 8, when the moving target is moving away from the imaging lidar system, the moving target is moving from near to far, and its color gradually changes from light cyan, which represents the near distance, to orange-red, which represents the far distance; at the same time, the stationary target does not undergo any changes.

The experimental results have fully demonstrated that the S-ERG method can be used to obtain the motion speed of the moving target in real time, and at the same time, it can also obtain the distance image of the moving target and the stationary target in real time, which greatly facilitates the application of the range-gated imaging lidar system.

## 4. Conclusions

In this paper, a measurement method for moving targets, the stepping equivalent range-gate method (S-SER method), is proposed. Using this method, the speed of the moving target can be obtained simultaneously on the basis of obtaining the distance information of moving and stationary targets at the front and back edges of the range gate. Experimental validation was carried out on the measurement of moving targets using the S-ERG method, and the validation results show that the delay step size does not affect the accuracy of the method in measuring the speed of moving objects. Compared to the actual speeds measured by the rotational displacement sensors, the error of the speed measurement is less than 5% for the targets moving at low and medium speeds. Subsequently, the S-ERG method is used to obtain the distance images of the stationary target and the moving target while simultaneously obtaining the speed of the moving target, which greatly extends the application range of the range-gated imaging lidar and has high practical value.

## Figures and Tables

**Table 1:** Measurements of target moving towards the lidar system by static ERG method.

Actual Speed (km/h)	Measured Speed (km/h)	Error (%)
11–12	11.30	1.74
13–14	13.83	2.4
15–16	15.69	1.23
19–20	19.44	0.3
22–23	22.67	0.76
28–29	28.95	1.58

**Table 2:** Measurements of target moving away to the lidar system by static ERG method.

Actual Speed (km/h)	Measured Speed (km/h)	Error (%)
9–10	9.83	3.47
11–12	11.65	1.30
13–14	13.84	2.52
20–21	19.97	2.58
24–25	23.18	5.39
27–28	27.38	0.44

**Table 3:** Measurements of target moving towards the lidar system by the S-ERG method with different delay step size.

Delay Step Size Is 1 ns			Delay Step Size Is 5 ns		
**Actual Speed (km/h)**	**Measured Speed (km/h)**	**Error (%)**	**Actual Speed (km/h)**	**Measured Speed (km/h)**	**Error (%)**
14–15	14.20	2.06	9–10	9.12	4.00
17–18	18.44	5.37	12–13	12.15	2.80
22–23	23.30	3.56	15–16	16.16	4.26
23–24	23.35	0.64	23–24	22.95	2.34
29–30	30.39	3.02	26–27	26.89	1.47
32–33	33.90	4.31	32–33	33.75	3.85

**Table 4:** Measurements of the target moving away to the lidar system by the S-ERG method with delay step size of 1 ns.

Actual Speed (km/h)	Measured Speed (km/h)	Error (%)
12–13	13.11	4.88
14–15	14.90	2.76
18–19	18.50	0
26–27	25.95	2.07
27–28	27.75	0.91
31–32	31.36	4.4

## Data Availability

Data are contained within the article.

## Abbreviations

The following abbreviations are used in this manuscript:

AFD	Adjacent frame difference
ERG	Equivalent range gate
S-ERG	Stepping equivalent range gate
3D	Three-dimensional
ICCD	Intensified-charge-coupled device
SNR	Signal-to-noise ratio
